# Impact of Particle Size Distribution in the Preform on Thermal Conductivity, Vickers Hardness and Tensile Strength of Copper-Infiltrated AISI H11 Tool Steel

**DOI:** 10.3390/ma16072659

**Published:** 2023-03-27

**Authors:** Johannes Vetter, Samuel Beneder, Moritz Kandler, Felix Feyer, Carolin Körner, Michael Schmidt

**Affiliations:** 1 Friedrich-Alexander-Universität Erlangen-Nürnberg, Institute of Photonic Technologies, Konrad-Zuse-Straße 3/5, 91052 Erlangen, Germany; samuel.beneder@outlook.de (S.B.); michael.schmidt@lpt.uni-erlangen.de (M.S.); 2 Erlangen Graduate School in Advanced Optical Technologies (SAOT), Paul-Gordan-Straße 6, 91052 Erlangen, Germany; 3 Institute of Advanced Materials and Processes (ZMP), Dr.-Mack-Straße 81, 90762 Fürth, Germany; carolin.koerner@fau.de; 4 Friedrich-Alexander-Universität Erlangen-Nürnberg, Materials Science and Engineering for Metals, Martensstraße 5, 91058 Erlangen, Germany; moritz.kandler@fau.de (M.K.); felix.feyer@fau.de (F.F.)

**Keywords:** spontaneous infiltration, metal matrix composites, tool steel, copper, thermal conductivity, tensile strength

## Abstract

Spontaneous infiltration of a porous preform by a metallic melt provides the potential of generating metal matrix composites (MMCs) with tailored combinations of material properties at low cost. The bulk of tool inserts for injection molding must sustain high mechanical and thermal loads and simultaneously exhibit high thermal conductivity for efficient temperature control of the mold insert. To fulfill these contradictory requirements, AISI H11 tool steel preforms were infiltrated by liquid copper. The impact of the fine powder fraction (0 wt.% to 15 wt.%) blended to a coarse H11 powder in the preform on thermal conductivity, Vickers hardness and tensile strength was elucidated. The thermal conductivity of the composites could be enhanced by a factor of 1.84 (15 wt.% fine powder) and 2.67 (0 wt.% fine powder) with respect to the sintered H11 tool steel. By adding 15 wt.% fine powder to the coarse host powder, the tensile strength and Vickers hardness of the copper-infiltrated steel were 1066.3 ± 108.7 MPa and 366 ± 24 HV1, respectively, whereas the H11 tool steel yielded 1368.5 ± 89.3 MPa and 403 ± 17 HV1, respectively. Based on the results obtained, an appropriate particle size distribution (PSD) may be selected for preform preparation according with the requirements of a future mold insert.

## 1. Introduction

Molds for injection molding are highly intricate industrial components with regard to the geometry, chemical stability and mechanical and thermal requirements of the mold material [1]. According to the state of the art, the tools are manufactured from tool steel, since its properties widely fulfill the material requirements and it is affordable. However, it is impossible to exactly meet the diverse requirement profile by one single material. Basically, a tool insert for injection molding may be classified into three main subdomains:

(i) Cavity:

Owing to the high pressure of the injected polymer melt, a good compression strength of the cavity material is mandatory. Moreover, particularly in the case of glass or carbon fibers admixed to the polymer the cavity material has to be wear-resistant [2]. Modern injection molds have to withstand up to several million cycles [3].

(ii) Cooling channels:

A sophisticated temperature control via conformal cooling channels reduces the cycle time and thus the efficiency of the injection molding process. A suitable material for the cooling channels must be corrosion-resistant and antibacterial [4,5].

(iii) Bulk:

The material between the cavity and the cooling channels, denoted as bulk, must withstand high mechanical loads and exhibit high thermal conductivity to enable good heat dissipation from the polymer melt and the cooling medium.

As pointed out, the outlined requirements can be met best when selecting the optimal material for each subdomain. With regard to the bulk, the commonly deployed tool steels (e.g., H11 or H13) are well suited for their good mechanical properties. However, the generally low thermal conductivity must be elevated [6], ensuring efficient temperature control of the injection mold insert. High thermal conductivity steels (HTCS), e.g., Rovalma HTCS-150, may be a better option from the perspective of thermal behavior. However, when used for hot press forming, they suffer from alternations of surface structure as a consequence of temperature changes [7] and are hard to machine with conventional technologies [8].

Spontaneous infiltration as a method of powder metallurgy may provide the option to generate interpenetrating phase composites (IPCs) with novel combinations of material properties [9]. Infiltration processes are driven by capillary forces drawing a metallic melt into a porous body [10]. This porous preform is prepared by presintering a powder compact to generate and maintain a specific shape of the reinforcing phase to be infiltrated subsequently by a lower melting material. Therefore, a composite consisting of H11 tool steel and copper could be a promising material for the bulk of a tool insert for injection molding. Hot work tool steels, e.g., H11 and H13, are widely used in mold construction due to their inherently good tensile strength, toughness and hardness [11,12,13]. Copper as an infiltrant is selected for its superior thermal conductivity [14] and advantageous infiltration behavior in combination with steels [15]. The latter is closely connected to the contact angle between liquid copper and the steel. In the case of a pure iron surface, the contact angle of liquid copper at a temperature of 1130 °C reduces with time from approx. 28 ° at t = 0 to approx. 13 ° at t > 15 min [16], which is equivalent to excellent wetting behavior. The H11/Cu metal matrix composite (MMC) is expected to show good mechanical properties and significantly enhanced thermal conductivity with respect to those of H11 tool steel.

The resulting physical and mechanical properties of the H11/Cu MMC are highly dependent on the topology and volume fraction of the constituents [17]. A promising way to control the structure of the reinforcing phase and thus numerous crucial properties of the composite after infiltration encompasses the variation of the PSD of the powder utilized for preform preparation [18]. In order to enlarge the residual porosity of the preform present for infiltration, Klein et al. [15] preferred a coarse, poorly sinterable PSD between 63 and 80 μm for the X245VCrMo9-4-4 steel. As a consequence, the fraction of copper in the composite was high and therefore the thermal conductivity was increased from 21.3 W/(mK) for the tool steel to 50.1 W/(mK) for the infiltrated composite. On the other hand, it may be expected that the mechanical properties of the MMC are moderate since sintering of the coarse powder particles results in poor interconnections of the particles providing diminished load-bearing capacity. Well-connected powder particles in the H11 preform may lead to increased tensile strength. Therefore, a mixture of predominantly coarse powder (>20 μm) dedicated for powder bed additive manufacturing and a minor fraction of fine, sinterable metal injection molding (MIM) powder (<15 μm) was used. This may result in sufficient residual porosity and also good interconnectivity of the powder particles in the preform after presintering.

To date, spontaneous infiltration is a common technique for the generation of IPCs in several fields of application. Cramer et al. [19] investigated a tungsten carbide-cobalt cermet composite manufactured by binder jet additive manufacturing and subsequent cobalt infiltration and proposed it for mining bits and complex-shaped cutting tools owing to its combination of high hardness and fracture toughness. Electrical contact materials exhibiting high electrical and thermal conductivity as well as good wear behavior at elevated temperatures were prepared by infiltration of copper-based infiltrants into tungsten [20] and tungsten carbide [21]. In the automobile industry, copper infiltration is used for valve seat inserts in combustion engines in order to elevate the thermal conductivity of the material [22,23]. Seleznev and Roy-Mayhew [24] developed a method to create a bi-metal composite consisting of tool steel (H13 and 17-4PH) and copper by printing a steel preform with a porous infill by fused filament fabrication (FFF). Subsequent to presintering, they infiltrated the millimeter-scale voids in the preform with liquid copper. The thermal conductivity was greatly enhanced from 18 W/(mK) in H13 tool steel to 108 W/(mK) in the bi-metal composite. Sachs et al. [25] employed a binder jet additive manufacturing technology to print a porous steel preform of a tooling insert for injection molding and infiltrated it with a bronze alloy. Considering surface mold temperature and stability, the infiltrated tool insert with conformal cooling channels demonstrated superior performance in the injection molding process with respect to conventionally drilled cooling channels. However, mechanical properties of the infiltrated composites destined for application in tooling are generally disregarded. Advanced tools employed in industrial processes have to sustain the applied mechanical loads for the lot size to be produced. Hence, fundamental knowledge of both, thermal and mechanical properties is mandatory to properly design a tool insert.

The present study aims at the development of H11/Cu MMCs by adding varying fractions of fine powder to a coarse, poorly sinterable H11 powder to prepare a porous H11 preform, followed by spontaneous copper infiltration. A systematic investigation as a function of the added fine powder fraction of inevitable material properties such as tensile strength, Vickers hardness and thermal conductivity may qualify the H11/Cu MMCs as an attractive option for the bulk material in injection molding tool inserts. These findings may allow a targeted selection of an appropriate PSD of the preform for proper tailoring of the MMC according to the requirements of a future mold insert.

## 2. Materials and Methods

### 2.1. Preform Preparation and Infiltration

**Figure 1:**
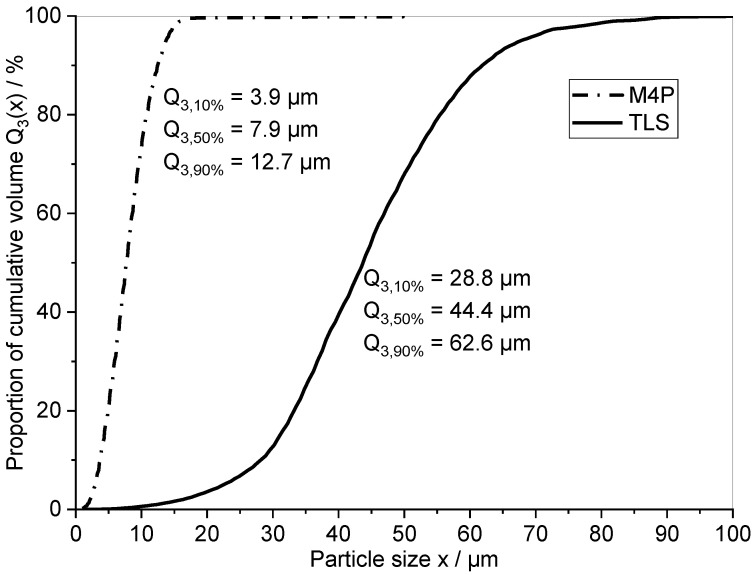
Powder particle size distribution (PSD) of the employed fine M4P H11 powder and the coarse TLS H11 powder.

Manufacturing H11/Cu composite materials encompasses the preparation of a porous skeleton by presintering H11 powder. Subsequently, the open porosity of the obtained preform is infiltrated by molten copper. The PSD of the powder used for preform preparation is believed to strongly affect the portion and structure of the retained porosity after presintering and thus the copper phase in the composite material. Hence, a fine H11 powder (M4P material solutions GmbH, Magdeburg, Germany) was blended to a coarse H11 powder (TLS Technik GmbH & Co. Spezialpulver KG, Bitterfeld, Germany) by using 0 wt.%, 5 wt.%, 10 wt.% and 15 wt.% of fine powder. Both powders showed a predominantly spherical shape. Figure 1 displays the PSD of the fine M4P powder and the coarse TLS powder determined by a Camsizer X2 particle analyzer (Retsch Technology, Haan, Germany). The y-axis labels the cumulative size distribution function *Q_3_(x)* representing the total volume or mass of powder particles below a certain particle size *x* [26]. Moreover, the characteristic values *Q_3,10%_*, *Q_3,50%_* and *Q_3,90%_* are given representing the particle sizes where 10%, 50% and 90% of the total cumulative volume of powder particles was reached. Table 1 provides the chemical composition of the employed H11 tool steel powders as specified by the manufacturers.

The powders were mixed for 2 h in a Turbula T2A tumbling mixer (Willy A. Bachofen AG, Muttenz, Switzerland). The blended powders were filled in alumina crucibles and slightly compacted by hand using a steel shaft. Accordingly, they were presintered at a maximum temperature of 1200 °C and a dwell time of 3 h at 1200 °C with a constant heating rate of 5 K/min in a HTK 25 MO/16 furnace (Carbolite Gero GmbH & Co. KG, Neuhausen, Germany) in vacuum at a pressure of 5 · 10^−6^ mbar. For infiltration, the presintered preforms again were put in alumina crucibles, and a piece of copper with a purity of ≥99.95% (Gemmel Metalle, Fürth, Germany) was placed on top of the preforms. At least 15% of excess copper material was used. Both, preform and copper were heated at a rate of 5 K/min to 1125 °C and held for 3 h at 1125 °C to melt the copper. The dwell time in the case of presintering was beneficial to forming strong sinter necks and ensuring complete penetration of the copper melt in the preform during infiltration. The heating rate on the one hand was limited by the moderate resistance to thermal shock of the employed alumina crucibles. Moreover, particularly large-scale components such as molding inserts must be heated slowly to avoid large temperature gradients in the component resulting in inhomogeneous shrinkage rates and thus cracking.

**Figure 2:**
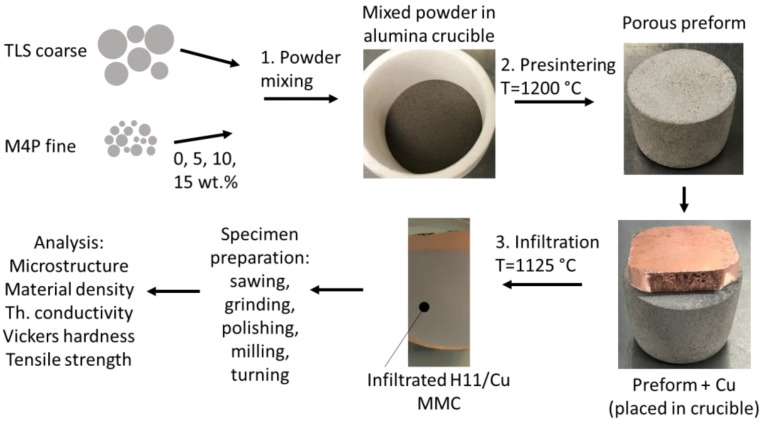
Preparation of H11/Cu MMCs, including the steps of powder mixing, presintering to obtain a porous preform and infiltration of the preform with molten copper.

Figure 2 visualizes the process sequence for the preparation of the preforms and the subsequent infiltration process with copper. To monitor the pore structure and fraction of the preforms as a function of the fine powder addition, preforms were generated by presintering. These preforms were also subjected to the heat treatment during infiltration but without copper addition. As a reference to the composite materials, a specimen completely consisting of H11 tool steel was generated by sintering exclusively the fine powder at 1250 °C for 6 h.

**Table 1:** Chemical composition in wt.% of the employed H11 tool steel powders.

	C	Si	Mn	Cr	Mo	V	Fe
min	0.30	0.80	0.25	4.80	1.10	0.30	bal.
max	0.41	1.20	0.50	5.50	1.50	0.50	

### 2.2. Material Characterization

For determining the retained porosity of the presintered material and the relative density of the Cu-infiltrated composite, the specimens were first prepared by cutting, grinding and polishing. The cross-sections were then investigated by optical microscopy using an Olympus BX53M optical microscope. Afterwards, approx. 6 × 5 mm^2^ wide sections of the specimens were analyzed by the image analyzing software Gimp 2.10 regarding their pixel brightness. A threshold of 170 was used for the 8-bit grayscale image in order to generate a binary image. The relative density was calculated as the ratio of the number of white pixels to the total pixel number.

The shrinkage of the preforms was determined by measuring the diameter of the employed alumina crucibles with an internal measuring gauge and the diameter of the sintered preforms with a digital caliper. For each PSD, the mean value and the 95% confidence interval out of three specimens are reported.

For energy-dispersed spectroscopy (EDS) elucidating the distribution of the elements in the H11/Cu composite, a Merlin Gemini 2 scanning electron microscope (Carl Zeiss, Oberkochen, Germany) equipped with an X-Max EDS detector (Oxford Instruments, Abingdon, United Kingdom) was used. The EDS was performed using a working distance of 8.5 mm and an acceleration voltage of 15 kV.

The material density for the manufactured composite materials was determined according to Archimedes’ method using deionized water and the surfactant Pervitro 75% as the immersion medium. Three specimens of 1.5 ± 0.2 g were cut from the infiltrated material for each preform PSD. A hydrostatic balance with a display accuracy of 0.01 mg was employed for the determination of the density.

Thermal diffusivity a was determined using an LFA 1000 laser flash system (Linseis Messgeräte GmbH, Selb, Germany). For laser flash analysis (LFA), the specimens were cut to dimensions of 10 × 10 × 1.5 mm^3^ (L × W × d). Thermal diffusivity a was calculated using Equation (1).

(1) $$a=1.38\frac{d^{2}}{\pi^{2}\cdot t_{0.5}}$$

Here, d represent the thickness of the specimen, and $t_{0.5}$ the time required to obtain 50% of the temperature increase caused by the laser pulse. The thermal conductivity λ of the composites and the H11 tool steel was calculated according to

(2) $$\lambda =a\cdot \rho \cdot c_{P}$$

where ρ denotes the material density, and $c_{P}$ the specific heat capacity. In order to cover typical mold temperatures in polymer injection molding, λ was determined for 25, 50, 85 and 120 °C.

The determination of Vickers hardness HV1 was conducted by means of a KB 30 S hardness-testing machine (KB Prüftechnik GmbH, Hochdorf-Assenheim, Germany). Prior to the measurement, the specimens were polished. For each specimen, nine measurements were carried out on the surface in a 2 × 2 mm^2^ wide section with a distance of 1 mm between the indentations, and the mean value and the 95% confidence interval are reported.

**Figure 3:**
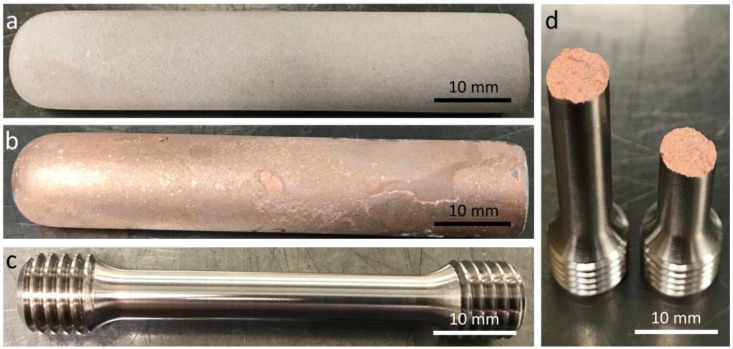
Stages in preparation and testing of the H11/Cu tensile testing specimens with (**a**) porous H11 preform, (**b**) copper infiltrated blank, (**c**) machined H11/Cu tensile specimen and (**d**) tensile specimens subsequent to tensile testing.

Tensile strength is crucial for injection molding tools, as they need to withstand high mechanical loads. Since the PSD in the preform strongly affects the structure of both, the reinforcing H11 phase and the copper matrix, a considerable impact on tensile properties is also expected. Therefore, tensile specimens were created out of each PSD from 0 wt.% to 15 wt.% of fine powder addition. The tensile properties were determined on both, the porous preforms and the infiltrated H11/Cu composite materials. The powders were filled in high cylindrical alumina crucibles and presintered to porous skeletons for 3 h at 1200 °C. Thus, cylindrical blanks of approx. 70 mm in length and 15 mm in diameter were obtained. For testing the preforms, the blanks were directly machined to tensile specimens according to DIN EN ISO 6892-1 with a diameter of 6 mm. To test the H11/Cu composites, the preforms were first infiltrated with Cu at 1125 °C (3 h) by placing a cylindrical piece of Cu on top of the preform. The H11/Cu composites were then turned to tensile specimens. Figure 3 displays the distinct stages during preparation of the H11/Cu tensile specimens. The tensile tests were performed using a Quasar 100 universal testing machine (Galdabini, Cardano al Campo, Italy) with a 100 kN load cell. The gauge length of the specimens was 30 mm, and the strain rate was set according to DIN EN ISO 6892-1. For each PSD of the preform, the mean values and 95% confidence intervals of three tensile specimens are reported. The fractured surfaces of the tensile specimens were imaged using a VHX-100 digital microscope in combination with a VH-Z20 objective (Keyence, Osaka, Japan) at 30× magnification.

## 3. Results and Discussion

### 3.1. Characterization of the Preforms

**Figure 4:**
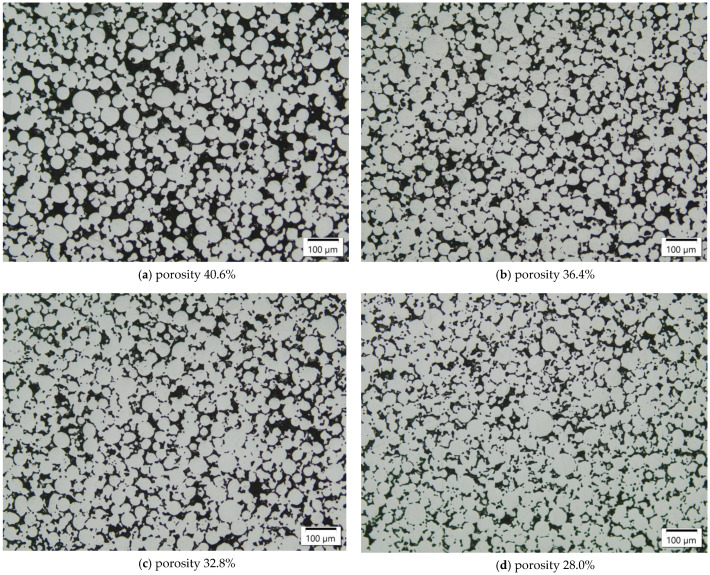
Pore structure for H11 tool steel preforms sintered for 3 h at 1200 °C with (**a**) 0 wt.%, (**b**) 5 wt.%, (**c**) 10 wt.% and (**d**) 15 wt.% fine powder addition, imaged by optical microscopy.

Most of the sinter-based approaches, including MIM and material extrusion additive manufacturing [27], aim for a completely dense microstructure subsequent to sintering. For infiltration, however, a high fraction of open porosity is required in the preform. Coarse powders exhibiting a low driving force for sintering provide sufficient porosity for the preform to be infiltrated. Nonetheless, additionally to the fraction, the structure of the reinforcing preform material is believed to strongly affect the material properties of the composite material. Therefore, it was intended to obtain soundly interconnected powder particles in the presintered preform by preparing a mixture of mainly coarse powder and a smaller amount of fine powder. Figure 4 illustrates the pore structures of the H11 preforms sintered at 1200 °C containing up to 15 wt.% of fine powder. The coarse powder without an addition of fine powder resulted in small necks formed between the powder particles but also the highest retained porosity present for infiltration. With the fine powder addition increasing, the powder particles were progressively coalesced, and the retained porosity decreased, since the fine powder particles occupied the interstices between the coarse particles. This was closely connected to the change in the bulk density of the powder mixtures from lowest for the pure coarse powder increasing with the amount of fine powder fraction added. The fine sinterable powder particles were no longer apparent as such but were sintered onto the coarse particles, leading to better interconnectivity and a finer structure of the remaining porosity.

In the future, the H11/Cu composite materials investigated in this work should be used for the bulk of tool inserts for injection molding. This may involve sinter-based additive manufacturing techniques, for instance, material extrusion as described in [28] or feedstock for photopolymerization [29]. In both cases, the feedstock has to be filled with suitable steel powder to prepare the porous preform of the tool insert. With the goal of achieving nearly full density, as is typically the case when sintering FFF- or MIM-based parts, linear shrinkage is 15% or more for solid loading of approx. 60 vol.% [30], and large-scale tool inserts are therefore prone to cracking during sintering. The infiltration-based approach, however, involves significantly less shrinkage for presintering the preform, since a large fraction of the porosity is retained for infiltration. Hence, the sintering process is believed to be less susceptible to part failure for infiltration with respect to conventional sintering. Table 2 lists the shrinkage values and tensile strength of the preforms as a function of the PSD subsequent to sintering at 1200 °C for 3 h. As it is very much straightforward, the overall linear shrinkage increased with the content of fine powder added. However, the shrinkage of all PSDs was well below the value with MIM, and thus presintering and infiltration may be capable of generating larger and more massive geometries without defects. Moreover, the shrinkage must be precisely monitored to meet the desired tolerances of the final component. A smaller overall shrinkage of a powder compact during sintering is believed to result in a better dimensional accuracy. The tensile strength for 0 wt.% and 5 wt.% fine powder additions did not differ significantly, higher fractions of fine powder increased the preform strength. Aside from the diminished retained porosity as a consequence of the fine powder, the enhanced tensile strength originated from extended sinter necks, providing more stable connections between the coarser particles.

### 3.2. Characterization of the H11/Cu Composites

**Figure 5:**
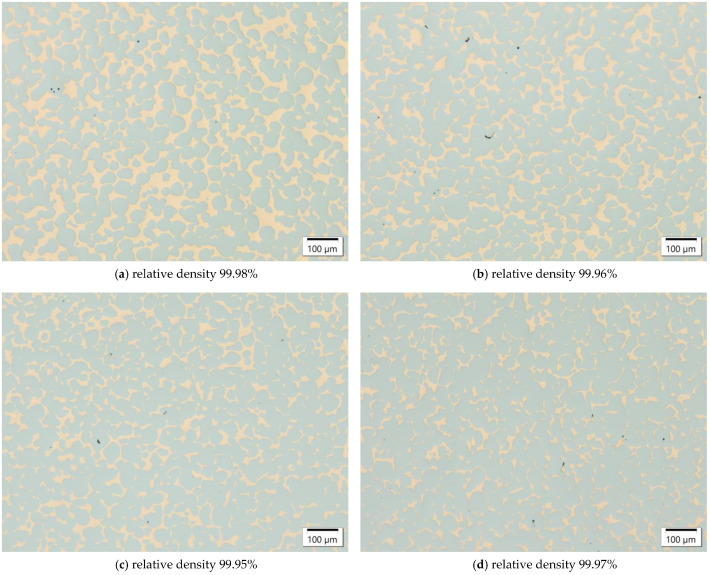
H11/Cu composite materials manufactured from H11 tool steel preforms with (**a**) 0 wt.%, (**b**) 5 wt.%, (**c**) 10 wt.% and (**d**) 15 wt.% fine powder contents due to infiltration with Cu at 1125 °C (3 h), imaged by optical microscopy.

Subsequent to presintering the tool steel powder, the open porosity of the preforms was infiltrated by liquid copper. The copper melt was drawn into the porous tool steel by capillary forces. Figure 5 displays the H11/Cu MMCs with fine H11 powder contents in the preforms from 0 wt.% to 15 wt.%. Since the residual open porosity in the H11 presintered preforms decreased with increasing fine powder content, the volume fraction of the copper decreased accordingly. The pore channels became narrower with increasing fine powder content, resulting in a generally finer structure of the copper phase in the H11/Cu composite material. The relative density was obtained by image analysis of the micrographs, and its procedure is described in detail in Section 2.2. As can be seen from the images, the copper melt in combination with the H11 preform skeleton exhibited favorable infiltration behavior, as the relative density of the infiltrated composites was well above 99.90% irrespective of fine powder content in the preform powder. Thus, the apparently good wettability of the copper melt in contact with the H11 preform allowed for a practically complete infiltration. For comparison, the relative density of the sintered H11 tool steel manufactured using the fine powder was found to be 95.70% due to residual porosity. Despite a discrepancy regarding the coefficient of thermal expansion of tool steel (approx. $12\cdot 10^{-6}{\;K}^{-1}$) and copper (approx. $17\cdot 10^{-6}{\;K}^{-1}$), the composite materials were free of cracks. The sinter necks of the H11 tool steel subsequent to infiltration were intact, which is crucial for good mechanical behavior.

The residual porosity of the composite materials can be either assigned to closed porosity at the moment of infiltration or to open porosity in the shape of acute pore channels being too sharp for infiltration. With regard to closed porosity, pores inside the powder particles as a consequence of the powder atomization process are most likely to occur. Furthermore, closed porosity may also arise by presintering the preform powder if a void is completely encapsulated so that the copper melt cannot access it. Even though the fine powder content increased, no clear trend could be observed with regard to encapsulated voids in the composite material.

**Figure 6:**
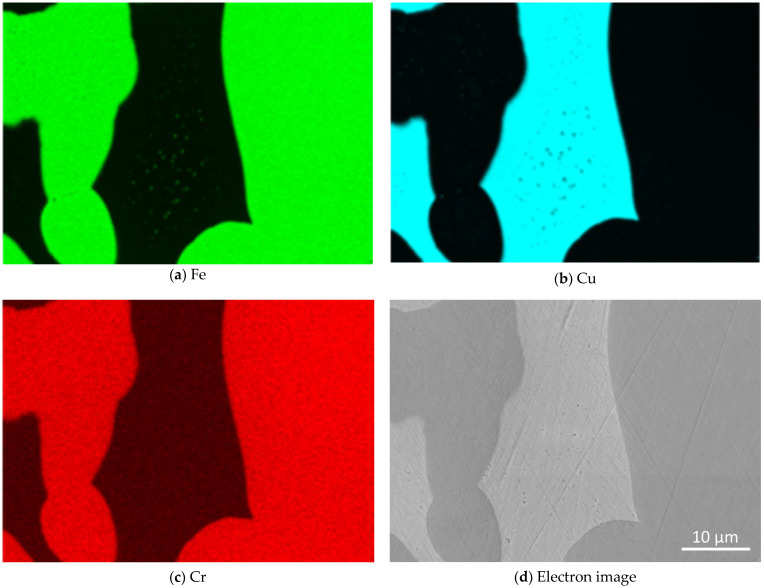
EDS images of the H11/Cu composite material with 15 wt.% of fine H11 powder showing the elemental distribution of (**a**) Fe, (**b**) Cu and (**c**) Cr as the alloying element with the highest concentration in H11; (**d**) the electron image.

In order to investigate the elemental distribution in the composite and evaluate the presence of intermetallic phases, EDS measurements were conducted. Figure 6 shows the mappings of the main elements, Fe, the infiltrant Cu and Cr as the alloying element in H11 with the highest concentration. Cu and the steel (Fe and Cr) phase are generally separated giving rise to absence of intermetallic phases. It was recognized before by Klein et al. [15] that iron has a solubility in liquid copper of approx. 3 wt.%. Dissolved iron formed precipitations in the copper matrix as it was cooled down. These precipitations are apparent in Figure 6a as small green areas of iron within the copper phase and can be seen clearly in Figure 6b as black points.

The volume fraction and the structure of each phase in a composite has a profound effect on their thermal and mechanical properties [17]. Besides the capability of generating conformal cooling channels by means of additive manufacturing, a further efficiency gain of injection molding tool inserts can be obtained by elevating the thermal conductivity of the mold material. Thermal diffusivity a and material density ρ of the H11/Cu composite materials are given in Table 3. The specific heat capacity $c_{P}$ was calculated using the rule of mixture for the constituents H11 and the copper matrix as proposed by Lindemann et al. [31]. According to [15], the copper phase in the infiltrated composite contains 3 wt.% of iron due to the solubility of iron in copper. Hence, the $c_{P}$ value of this alloy (423 J/kgK [15]) was used, and the $c_{P}$ of the H11 steel is 460 J/kgK [32]. These data are used to calculate the thermal conductivity of the H11 and H11/Cu composites according to Equation (2). The thermal diffusivity was determined for 25, 50, 85 and 120 °C. This temperature interval contains typical mold temperatures for a vast number of injection-molded polymers. With the copper volume fraction in the MMC increasing, thermal diffusivity was significantly enhanced. Material density was determined according to Archimedes’ method. Since the content of copper (density 8.9 g/cm^3^) in the composite decreased with increasing the fine powder addition of H11 (density 7.8 g/cm^3^) in the preform, material density was reduced from 8.10±0.05 g/cm^3^ for 0 wt.% fine powder to 7.99±0.02 g/cm^3^ for 15 wt.% fine powder addition.

**Figure 7:**
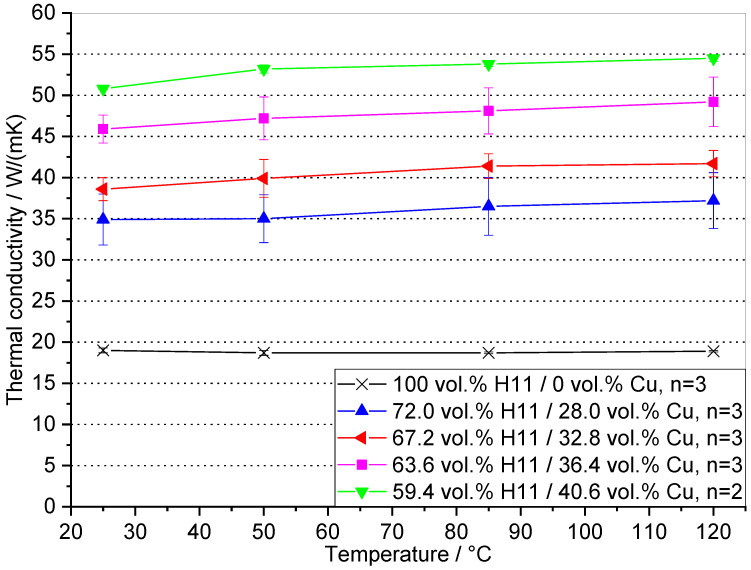
Thermal conductivity λ of the four manufactured H11/Cu composite materials and the sintered H11 tool steel as a function of temperature.

The thermal conductivity of the H11 tool steel and the H11/Cu composite materials was calculated using the values for thermal diffusivity, material density and specific heat capacity presented in Table 3. According to Figure 7, the thermal conductivity of the H11 tool steel was moderate and thus would compromise efficient temperature control for injection molding tool inserts. Since with decreasing fraction of fine powder used to prepare the composites the fraction of copper phase was elevated, thermal conductivity was enhanced accordingly. Klein et al. [17] found that the steel phase contributes to 1/6 and copper to 5/6 to the overall thermal conductivity for X245VCrMo9-4-4 copper-infiltrated tool steel. For the H11/Cu MMCs, the temperature had a weak effect on thermal conductivity between 25 and 120 °C. On the other hand, the composition of the composites had a significant impact on thermal conductivity and could be elevated at 25 °C by a factor of 1.84 (72.0 H11/28.0 Cu) to 2.67 (59.4 H11/40.6 Cu) with respect to the H11 tool steel.

The measurements of thermal diffusivity were conducted within a temperature interval covering typical tool temperatures for the majority of technically relevant polymers. The findings for the pure coarse powder with regard to thermal conductivity (50.8 W/(mK)) are in good concordance with those of the study of Klein et al. [15] where 50.1 W/(mK) was obtained in the aged state. As the composites in this study were not quenched, precipitations of iron were present in the copper matrix. They were also found in [15,24], resulting from diminished solubility of iron in copper with decreasing temperature.

For tooling, the hardness, tensile strength and elongation at break are among the most important mechanical properties [33]. Therefore, this section deals with these properties as a function of the fine powder content in the H11 preform. These findings are finally relevant for construction to design the mold insert and select an appropriate PSD of the preform based on the requirements of thermal conductivity and mechanical loads.

**Figure 8:**
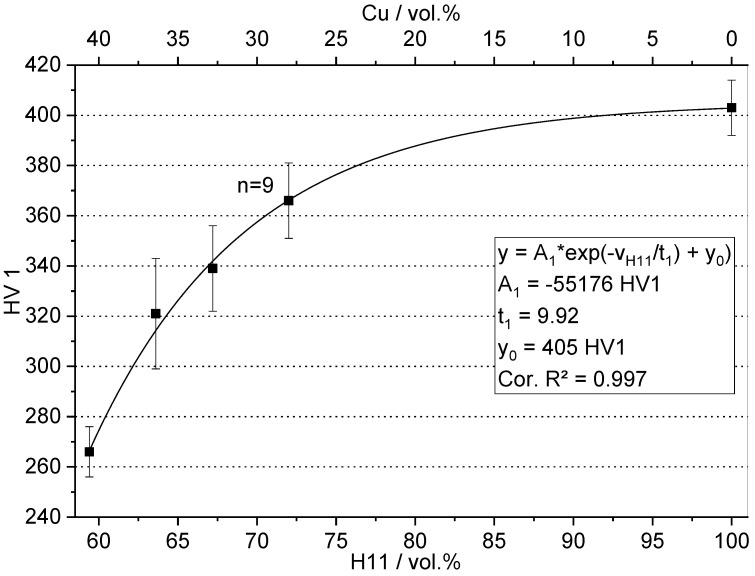
Vickers hardness HV1 of the H11/Cu composite materials and H11 as a function of H11 content in the as-infiltrated/as-sintered state.

Vickers hardness HV1 in the as-infiltrated (H11/Cu) or as-sintered state (H11) is depicted in Figure 8. For each data point, the mean value and the 95% confidence interval of nine measurements are reported. The distinct fractions of H11 and Cu were obtained by fine powder addition. From left to right, the data points correspond to 0 wt.%, 5 wt.%, 10 wt.%, 15 wt.% and 100 wt.% fine powder in the preform. High contents of soft copper matrix in combination with poor connections of the H11 tool steel sinter necks yielded moderate Vickers hardness. With H11 content increasing, the hardness was enhanced continuously due to the inherently harder tool steel and more stable sinter necks formed by the fine powder during presintering of the preform. The pure H11 tool steel specimen sintered from 100 wt.% fine H11 powder exhibited a hardness of 403 HV1. The data points were fit by a first-order exponential function by which Equation (3) was obtained.

(3) $$\mathrm{Vickers}\;\mathrm{hardness}=-55176\;\mathrm{HV}1\cdot \exp\left(-\frac{v_{H11}}{9.92}\right)+405\;\mathrm{HV}1$$

In this context, $v_{H11}$ represents H11 content (in vol.%). It is important to note that the fit function does not allow to extrapolate the expected Vickers hardness of H11 contents below the lowest content of 59.4 vol.% investigated corresponding to 0 wt.% fine powder addition. As can be recognized from Figure 8, the data points correlated well with the fit function (Cor. R^2^ = 0.997). Sufficient iterations were performed so that the fit function converged. Hence, Equation (3) could be employed to calculate hardness values for arbitrary H11 contents between 59.4 vol.% and 100 vol.%. Typically, the hardness of tool steel in service when used for injection molding is around 52 HRC [34] (approx. 550 HV), which is attained by austenitizing, quenching and tempering. Therefore, the hardness values accomplished for both the H11/Cu MMCs and the H11 tool steel were not yet sufficient for construction of persistent mold inserts. Proper heat treatment aimed at secondary hardening of the H11 tool steel is believed to elevate the hardness of the composites, which is to be examined in a future study.

Taking into account the rule of mixture, the fit function should follow a linear correlation. For Vickers hardness, the fit function obviously exhibited strong exponential behavior. This observation may be linked to the structure of the H11 tool steel and copper phase in the composite material. The coarse powder without addition of fine powder resulted in small, punctual sinter necks between the tool steel particles after presintering. Consequently, the tool steel phase in the composite material was poorly interconnected. During hardness testing, the punctual sinter necks seemed to be sheared off and pushed into the soft copper matrix. Thus, the properties of the composite were dominated by the copper phase to a higher extent, as would be the case with a thoroughly interconnected H11 phase at a comparable volume content. With fine powder content increasing, the sinter necks were more prominent. The hardness of the composite in this case was enhanced by the higher H11 volume fraction and better connections of the tool steel phase. Both cumulative effects are believed to result in the exponential behavior of Vickers hardness.

**Figure 9:**
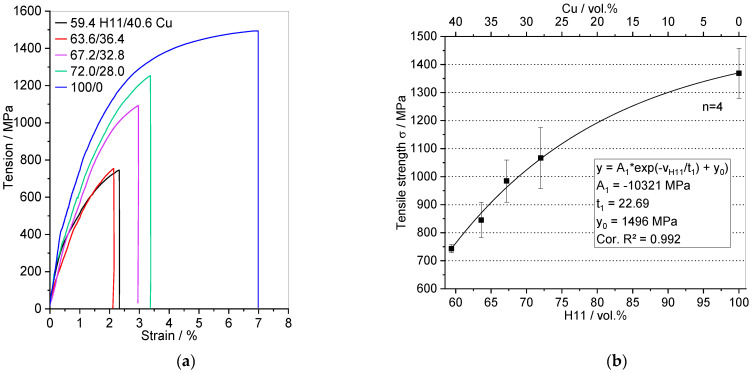
(**a**) Stress-strain curves and (**b**) tensile strength of the H11/Cu composite materials obtained by infiltration and the sintered H11 tool steel as a function of H11 content.

The stress-strain curves and tensile strength of the H11/Cu composite materials and the sintered H11 tool steel are depicted in Figure 9 in the as-infiltrated and as-sintered state, respectively. For each composition, one stress-strain curve is displayed. With an increasing amount of H11 tool steel, tensile strength and elongation at break were elevated. In Figure 9b, the data points represent, from left to right, 0 wt.%, 5 wt.%, 10 wt.%, 15 wt.% and 100 wt.% fine powder in the preform. Tensile strength was enhanced due to increasing H11 content in the composite and higher fine powder content resulting in more stable interconnections between the H11 powder particles in the preform after presintering.

In the case of tensile strength, exponential behavior was less pronounced with respect to that of Vickers hardness. Particularly the H11/Cu composite materials exhibited brittle fracture without any visible plastic deformation, as can be seen in Figure 3d. The fit function of Equation (4) could be used to calculate the tensile strength for any H11 content between the investigated range of 59.4 vol.% and 100 vol.%. The fit function was in good accordance with the measured values (Cor. R^2^ = 0.992), and the fit converged.

(4) $$\sigma =-10321\;\mathrm{MPa}\cdot \exp\left(-\frac{v_{H11}}{22.69}\right)+1496\;\mathrm{MPa}$$

**Figure 10:**
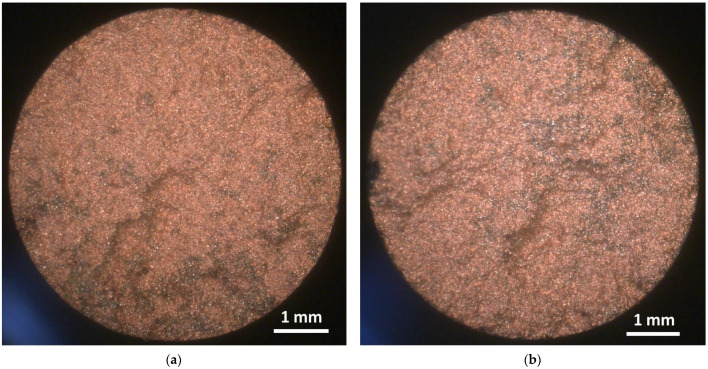
Optical images of the fractured surfaces of H11/Cu tensile specimens containing (**a**) 59.4 vol.% H11 and 40.6 vol.% Cu and (**b**) 72.0 vol.% H11 and 28.0 vol.% Cu.

In order to elucidate the fracture behavior of the H11/Cu composite materials, the fractured surfaces of the tensile specimens were analyzed. Figure 10 displays the fractured surfaces of the composites manufactured from preforms containing (a) 0 wt.% and (b) 15 wt.% of fine powder. From the fractured tensile specimens, there was no apparent necking, and the fractured surfaces gave rise to a predominantly brittle fracture. Both images of the fractured surfaces showed a disproportionally high amount of copper. This was a clear indication that the crack initiating fracture mostly ran through the copper phase. For the H11 tool steel phase, the weakest points, i.e., the sinter necks, were fractured. This conclusion was also supported by the increase in elongation at break for composites with high fine powder addition. Consequently, this fact underlines the significance of strong interconnections of the H11 preforms by adding fine powder to form strong sinter necks.

Considering the afore mentioned effects of PSDs in the starting powder and thus the copper fraction in the composites on tensile strength, Vickers hardness and thermal conductivity, a suitable PSD may be selected for the construction of a tool insert. In the case of a tool requiring high thermal conductivity and lower mechanical properties, a high fraction of copper in the composite is beneficial, and vice versa. In this context, the mechanical requirements of a tool are more critical than the thermal properties, since the tool has to reliably withstand the applied mechanical loads for its entire lifetime.

## 4. Conclusions

In this study, the impact of PSD in the starting powder on the properties of copper-infiltrated H11 tool steel was investigated. Fine powder was blended to a coarse H11 powder by mixing ratios of 0 wt.% to 15 wt.%, and the H11 preform obtained after presintering was infiltrated by liquid copper. Comprehensive analyses of the resulting material properties were conducted. The main findings may be summarized as follows:

▪ The shrinkage of the preforms was found to be less than half with respect to that in the MIM or FFF approach. When sintering large-scale and massive components, as is required for the majority of tool inserts, the infiltration-based approach is therefore believed to be less susceptible to thermal cracking. Hence, the process limitation of the conventional sintering process may be shifted towards larger components with higher mass.
▪ For all specimens regardless of PSD, practically complete infiltration of the preforms was observed. Consistently accomplished relative densities of more than 99.90% gave rise to the infiltration process being robust and thus well suited for the production of industrially relevant high-performance components.
▪ By varying the addition of fine powder in the starting powder, the thermal conductivity, material hardness and tensile strength could be varied within a wide range. With respect to the sintered tool steel, thermal conductivity was elevated for the composites by a factor in the range of 1.84 to 2.67 with simultaneously compromised mechanical properties. With the thermal and mechanical requirements known, a certain tool insert can be manufactured using the appropriate PSD that can be calculated by the determined fit functions.

## Figures and Tables

**Table 2:** Porosity, linear shrinkage (n = 3) and tensile strength (n = 3) of the preforms sintered from 0 to 15 wt.% fine M4P powder added to coarse TLS powder.

TLS/wt.%	M4P/wt.%	Porosity/%	Shrinkage/%	Tensile Strength σ/MPa
100	0	40.6	5.42 ± 0.15	161.0 ± 27.7
95	5	36.4	5.86 ± 0.39	168.1 ± 9.5
90	10	32.8	6.14 ± 0.25	257.7 ± 10.9
85	15	28.0	7.12 ± 0.24	370.5 ± 25.1

**Table 3:** Thermal diffusivity a, material density ρ and specific heat capacity $c_{P}$ for the H11/Cu composite materials and the sintered H11 tool steel.

TLS/wt.%	M4P/wt.%	Cu/vol.%	a/cm^2^/s				ρ/g/cm^3^	$c_{P}$/J/(kg·K)
			25 °C *	50 °C *	85 °C *	120 °C *	RT	
100	0	40.6	$0.141\left(1\right)$	$0.148\left(2\right)$	$0.149\left(1\right)$	$0.151\left(1\right)$	$8.10\left(5\right)$	445 [15,32]
95	5	36.4	$0.127\left(5\right)$	$0.131\left(7\right)$	$0.133\left(8\right)$	$0.137\left(8\right)$	$8.07\left(1\right)$	447 [15,32]
90	10	32.8	$0.108\left(4\right)$	$0.111\left(6\right)$	$0.115\left(4\right)$	$0.116\left(5\right)$	$8.01\left(1\right)$	448 [15,32]
85	15	28.0	$0.097\left(9\right)$	$0.097\left(8\right)$	$0.102\left(10\right)$	$0.103\left(9\right)$	$7.99\left(2\right)$	450 [15,32]
0	100	0	$0.056\left(1\right)$	$0.055\left(1\right)$	$0.055\left(1\right)$	$0.055\left(1\right)$	$7.42\left(6\right)$	460 [32]

* The measurement of thermal diffusivity was conducted within ±8 K around the given set value. The numbers in parentheses represent the 95% confidence interval for the last digits, i.e., $0.097\left(9\right)$ is equivalent to 0.097±0.009.

## Data Availability

The data presented in this study are available upon request from the corresponding author.
